# What works best for whom? Cognitive Behavior Therapy and Mindfulness-Based Cognitive Therapy for depressive symptoms in patients with diabetes

**DOI:** 10.1371/journal.pone.0179941

**Published:** 2017-06-29

**Authors:** K. Annika Tovote, Maya J. Schroevers, Evelien Snippe, Paul M. G. Emmelkamp, Thera P. Links, Robbert Sanderman, Joke Fleer

**Affiliations:** 1 Department of Health Psychology, University of Groningen, University Medical Center Groningen, Groningen, The Netherlands; 2 Interdisciplinary Center Psychopathology and Emotion regulation (ICPE), University of Groningen, University Medical Center Groningen, Groningen, The Netherlands; 3 Department of Clinical Psychology, University of Amsterdam, Amsterdam, The Netherlands; 4 HSK group, Woerden, The Netherlands; 5 Department of Endocrinology, University of Groningen, University Medical Center Groningen, Groningen, The Netherlands; 6 Department of Psychology, Health and Technology University of Twente, Enschede, The Netherlands; University of Glasgow, UNITED KINGDOM

## Abstract

**Objective:**

Cognitive Behavior Therapy (CBT) and Mindfulness-Based Cognitive Therapy (MBCT) have shown to be effective interventions for treating depressive symptoms in patients with diabetes. However, little is known about which intervention works best for whom (i.e., moderators of efficacy). The aim of this study was to identify variables that differentially predicted response to either CBT or MBCT (i.e., prescriptive predictors).

**Methods:**

The sample consisted of 91 adult outpatients with type 1 or type 2 diabetes and comorbid depressive symptoms (i.e., BDI-II ≥ 14) who were randomized to either individual 8-week CBT (n = 45) or individual 8-week MBCT (n = 46). Patients were followed for a year and depressive symptoms were measured at pre-treatment, post-treatment, and at 9-months follow-up. The predictive effect of demographics, depression related characteristics, and disease specific characteristics on change in depressive symptoms was assessed by means of hierarchical regression analyses.

**Results:**

Analyses showed that education was the only factor that differentially predicted a decrease in depressive symptoms directly after the interventions. At post-treatment, individuals with higher educational attainment responded better to MBCT, as compared to CBT. Yet, this effect was not apparent at 9-months follow-up.

**Conclusions:**

This study did not identify variables that robustly differentially predicted treatment effectiveness of CBT and MBCT, indicating that both CBT and MBCT are accessible interventions that are effective for treating depressive symptoms in broad populations with diabetes. More research is needed to guide patient-treatment matching in clinical practice.

## Introduction

Depression and diabetes are common coexisting conditions that have a debilitating impact on each other [1]. As diabetes not only negatively affects patients’ physical health but also their mental health, a substantive number of patients suffers from depressive symptoms [2,3]. Cognitive Behavior Therapy (CBT) is the most commonly used evidence-based psychological intervention for treating depressive symptoms in individuals with and without a chronic somatic disease [4–6]. In the past decade, Mindfulness-Based Cognitive Therapy (MBCT) has evolved as an upcoming therapy for treating depressive symptoms [7]. Although the field of research on MBCT for current depressive symptoms is still relatively young, existing studies have provided consistent empirical support for the effectiveness of MBCT in reducing depressive symptoms and increasing quality of life in healthy as well as in chronically ill people [8–10].

CBT and MBCT can be characterized as variants of cognitive therapy, but they do include fundamentally different treatment techniques. CBT is a structured program that focuses on behavioral activation and on helping patients to understand connections among thoughts, feelings, and behaviors in order to consequently alter underlying cognitions that maintain a depressed mood [11]. MBCT, on the contrary, involves practicing awareness and acceptance of dysfunctional thoughts, feelings, and bodily sensations by focusing on the present moment during mindfulness meditation and yoga exercises [7]. Because of the different focus of CBT and MBCT, it could be that these treatments are beneficial for different groups of patients.

A few studies have directly compared the effects of CBT and mindfulness-based interventions [12–15]. Those studies provided evidence for the effectiveness of both interventions with respect to improving a variety of psychological and medical outcomes. In a recent randomized controlled trial, we compared the effects of individual CBT and MBCT in reducing depressive symptoms in comparison to a waiting list control condition in patients with diabetes [16]. Both interventions were found to be effective in reducing depressive symptoms, diabetes-related distress, and anxiety as well as in increasing well-being, with no intervention being superior over the other. Positive effects sustained at long-term follow-up at nine months after treatment [17].

This evidence, suggesting comparable effectiveness of both CBT and MBCT, does not necessarily imply that every individual patient benefits equally from these interventions. Characteristics of the individuals presenting for treatment are assumed to impact the level of treatment efficacy [18,19]. The current study investigates factors that predict differential response to CBT and MBCT.

Much of the literature on predictors of treatment efficacy has focused on investigating general prognostic predictors of CBT in order to identify subpopulations of patients that are most likely to benefit from CBT [20,21]. Typically, these studies have investigated predictors grouped in four different domains, namely demographics, clinical characteristics, personality dimensions, and disease related characteristics in somatic populations. Although demographics as predictors for CBT outcome have widely been studied, results are inconclusive as to which characteristics are of influence. Whereas some studies identified age [22], gender [23,24], education [22,23], marital status [22,25] as predictors, other studies failed to identify any demographic predictors [26–28]. In contrast, clinical characteristics such as high levels of pretreatment depressive symptoms, chronicity, and history of depression have consistently been associated with poorer CBT treatment outcome [20,27,29]. Studies that investigated personality dimensions as possible predictors of outcome mainly focused on neuroticism [30]. Again, no clear pattern emerged in the literature. Although high levels of neuroticism were associated with poor CBT outcome in some empirical studies [30–32], an earlier review by Mulder et al. [30] concluded that the influence of neuroticism firmly depended on the methodology of the study as the strongest support for the association came from methodologically weaker studies. Finally, disease-related variables like type of treatment, comorbidity, and diabetes-related distress may also predict outcome to psychological treatment. Lustman et al. [33] found that CBT was overall effective in reducing depressive symptoms, but that patients scoring lower on compliance with self-monitoring of blood glucose (SMBG) and with presence of diabetes complications achieved less remission of depressive symptoms than did patients with higher compliance with SMBG and no diabetes complications. Accordingly, diabetes symptom burden or diabetes-related distress might interfere with depression treatment and thereby negatively influence its effectiveness.

Comparatively, fewer studies have been conducted examining potential predictors of MBCT outcome and of these studies, the majority focused on recurrently depressed patients in remission. Kuyken et al. [34], investigated several possible general prognostic predictors like demographics, severity of depression, and depression recurrence and found that only gender was a significant predictor. Female participants showed a greater reduction in depressive symptoms at 15-months follow-up as compared to males. Yet, as the number of males in the study was low, the result should be replicated before drawing firm conclusions. In addition, another study by Nyklicek et al. [35] also found that females showed larger decreases in depression after MBCT as compared to males. Prior research by Teasdale and colleagues [36,37] reported that for patients with three or more previous depressive episodes, MBCT significantly decreased the risk of relapse, whereas this was not the case for participants with fewer episodes. Another study focusing on personality factors as predictors found that students had more benefit from Mindfulness-Based Stress Reduction when scoring high on neuroticism [38]. Hence, given the scarcity of previous research, firm conclusions about prognostic indicators of response to MBCT cannot be drawn.

Identifying general prognostic predictors within a given treatment may provide indications about who benefits most from that treatment, yet it offers no information on which type of treatment is more effective for a patient with certain characteristics. In this context, it is important to consider the interaction between patient characteristics and treatment modality by comparing active treatments with each other. The identification of those so-called prescriptive predictors, or moderators, can indicate subpopulations that fare better with one treatment than the other. Gaining insight into which treatment works best for whom can enhance optimal treatment selection, resulting in optimized clinical decision making and a more personalized care.

To date, only three RCTs investigated prescriptive predictors of CBT and mindfulness-based interventions. Manicavasagar et al. [14], investigating the effectiveness of MBCT and CBT for individuals with major depressive disorder, revealed differential effects for history of depression. CBT was more effective for individuals with four or more episodes, while MBCT was equally effective irrespective of the number of previous episodes. Arch et al. [13], although not focusing on depression but on individuals with anxiety, found that severity of current depressive symptoms was a moderator of effectiveness: individuals with no to mild depression had more benefit from CBT, whereas people with moderate to severe depression fared better with the mindfulness intervention. In another study, Zautra et al. [15] compared the effects of CBT and a mindfulness intervention to an education group with respect to several psychological and pain-related outcomes in a non-clinical sample of patients with rheumatoid arthritis. It was revealed that patients with recurrent depressive symptoms had most benefit from the mindfulness intervention. Unfortunately, these studies focused on clinical characteristics only, while other characteristics such as demographics, disease-related characteristics, and personality may also differentially predict change after treatment [18].

To advance the knowledge of prescriptive predictors (i.e., moderators) of the effects of CBT and MBCT, the current study investigated a broad range of patient characteristics to identify prognostic and prescriptive predictors in CBT and MBCT for depressive symptoms in patients with diabetes. We examined clinical meaningful patient characteristics that can be easily assessed by clinicians, namely demographics, clinical characteristics, neuroticism, and diabetes related characteristics. First, we examined which patient characteristics differentially predicted response to CBT and MBCT. Second, we investigated potential prognostic predictors separately for CBT and MBCT. While no clear hypotheses were formulated with respect to demographic, clinical, and disease specific characteristics, we hypothesized that patients scoring high on neuroticism will benefit more from MBCT as compared to CBT.

## Materials and methods

### Study design

The present study was embedded in a longitudinal multi-center, randomized controlled trial in which CBT and MBCT were compared to a waiting list control condition in terms of their effectiveness in reducing depressive symptoms (NCT01630512, ClinicalTrials.gov; the study was not registered before participants were included as it was then not mandatory to register non-drug intervention studies in the Netherlands. We registered the study immediately after we were notified of its importance). The study was approved by the Medical Ethical Committee of the University Medical Center Groningen (UMCG) on May, 18^th^ 2011 (S1 File) and compiled with the declaration of Helsinki and the CONSORT statement (S2 File). For an extensive description of the study procedures, the reader is referred to the original publication of the study protocol [39]. The authors confirm that all ongoing and related trials for this intervention are registered.

### Participants

Included participants were 18–70 years old with type 1 or 2 diabetes diagnosed at least three months prior to inclusion who had depressive symptoms as assessed with the BDI-II (cut off score ≥ 14 indicating the presence of at least mild symptoms of depression). Patients were excluded if they were not able to read and write, were pregnant, had a severe psychiatric comorbidity (including acute suicidal ideations), were receiving an alternative psychological treatment, or had started or altered their dosage of antidepressant drug within two months prior to participation.

### Procedure

**Fig 1:**
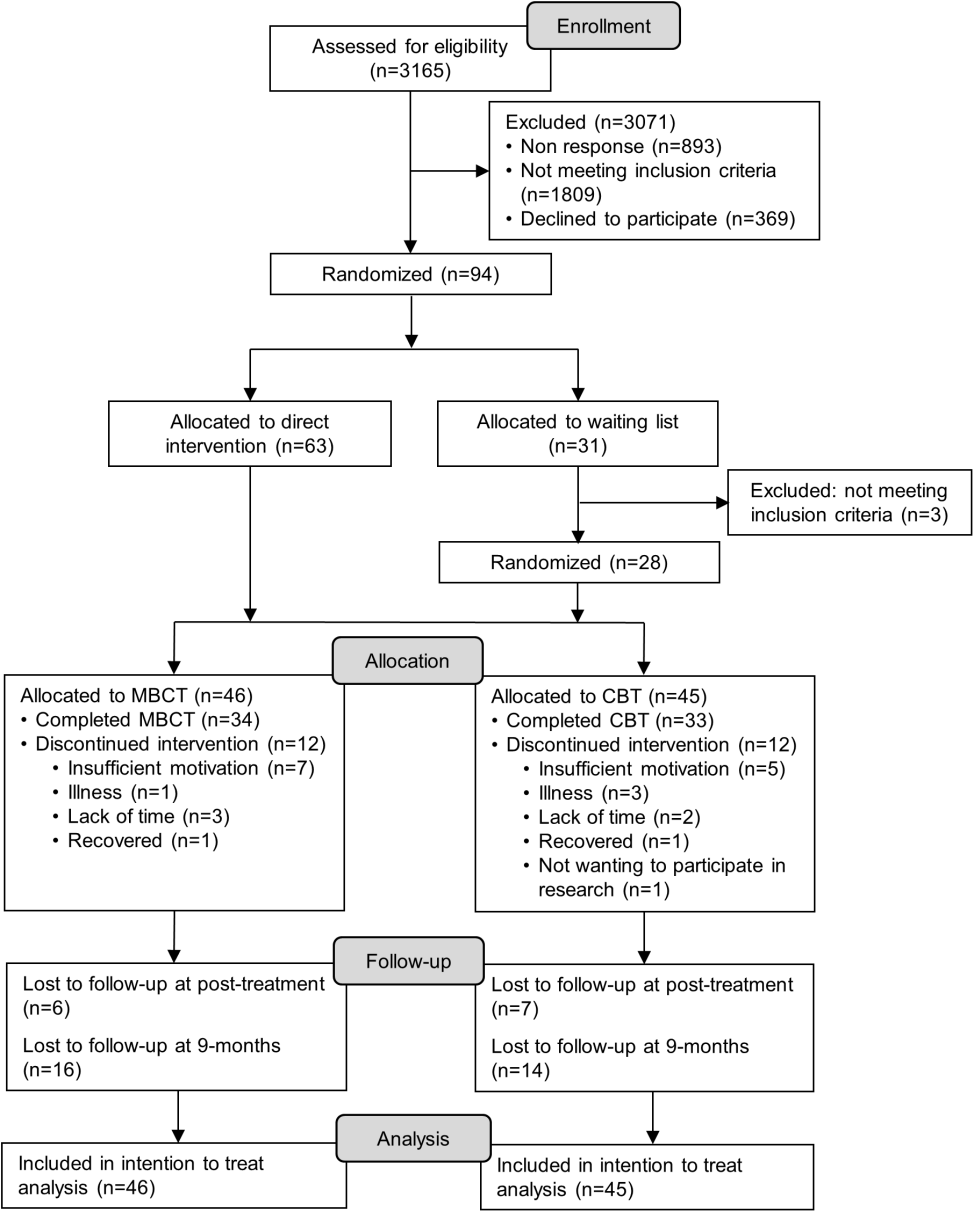
Flow of the study.

Patient recruitment took place from June 2011 to February 2013 and the last follow-up was concluded in April 2014. The trial was conducted in the Netherlands and included 94 participants. The patient flow is shown in Fig 1. Patients were recruited through standard screening at somatic outpatient clinics, referral by physician, and self-referral. In case of elevated levels of depressive symptoms, patients were invited for an intake during which they were assessed for eligibility. When criteria were met and patients gave written informed consent for participation, they were included in the study. Computerized randomization was carried out stratified by gender, use of antidepressant medication, and baseline BDI-II score. Before randomization, patients were blinded for the treatment condition. Consequently, patients were only told that they were to be randomized to a psychological treatment for reducing depression, starting within three months after randomization. Researchers performed the randomization and informed the participants. Following randomization, participants received individual CBT (n = 32), individual MBCT (n = 31), or were allocated to a waiting list control condition (n = 31). Patients allocated to the control group were randomized for the second time at the end of the three-months waiting period and received either CBT (n = 15) or MBCT (n = 16). For the purpose of the present study, participants in the waiting list condition were included in the analyses on condition that patients still had elevated levels of depressive symptoms after the waiting period (i.e., BDI-II ≥ 14). Three of the 31 patients did not score above the cut-off and were excluded, resulting in a total sample of 91 participants.

### Treatments

CBT and MBCT consisted of eight weekly outpatient treatment sessions of 45 to 60 minutes. Treatment was delivered individually by trained therapists that received supervision during the study period. Patients were expected to spend about 30 minutes per day on homework assignments. The CBT manual was based on CBT for depression developed by Beck [11]. The length of the training was shortened to eight sessions, to match the setting of the two treatments and to ensure a fit with the Dutch healthcare system. The MBCT treatment manual was based on the protocol developed by Segal, Teasdale and Williams [7]. As this protocol is developed as a 2.5-hour per session group treatment, we developed a shortened and individualized version of this protocol. This protocol has previously been tested in a pilot study in patients with diabetes and was evaluated as feasible and acceptable [40].

### Outcome measure

The primary outcome measure was change in depressive symptoms, assessed with the Beck Depression Inventory-II (BDI-II) [41] at pre-treatment, post-treatment (i.e. on average three months after pre-treatment), and at 9-months follow-up (i.e. on average 12 months after pre-treatment). The BDI-II consists of 21 items that are scored on a four-point scale from 0 (“not at all”) to 3 (“most of the time”). The items assess symptoms of depression such as sadness, loss of interest, and hopelessness during the last two weeks. Item scores are summated with a maximum score of 63. A BDI-II score of ≥ 14 indicates mild depressive symptoms. The reliability of the BDI-II was good in the current study (range α = 0.84–0.94).

### Predictor variables

Following the recommendations of Fournier et al. [22], we analyzed prospective predictors in different domains. All potential predictors and moderators of change were assessed before randomization at pre-treatment. Data were obtained from self-report questionnaires, intake interviews, and patients’ medical records.

#### Domain 1: Demographic characteristics

The following socio-demographic characteristics were included in the first domain: age (continuous), gender (dichotomous; (0) male, (1) female), education (three levels: lower level vocational school, secondary education/advanced level vocational school, higher or university education, coded into two dummy variables; (1) low levels of education, (2) high levels of education), employment (dichotomous; (0) unemployed, (1) employed), and marital status (dichotomous; (0) not in a relationship, (1) in a relationship). All variables were assessed by means of self-report.

#### Domain 2: Clinical characteristics and personality

The second domain consisted of current major depression, history of depression, history of psychological care, and neuroticism. Current major depression and history of depression were assessed during the intake by means of the depression section of the Structured Clinical Interview for DSM-IV (SCID-I) [42]. The output of the SCID was recorded as the presence (coded as 1) or absence (coded as 0) of a current or a past depressive episode. History of psychological care (i.e., psychologist or psychiatrist) was also assessed during the intake (dichotomous; (0) no history of care, (1) history of care). As neuroticism is strongly related to characteristics of depression, it was included in this domain. Neuroticism was assessed as a continuous variable by the subscale of the NEO- Five Factor Inventory (NEO-FFI) [43]. The internal consistency was good in the current study (α = 0.78).

#### Domain 3: Disease specific characteristics

The final domain, disease specific characteristics, included type of diabetes (dichotomous; (1) type I, (2) type II), treatment regimen (dichotomous; (0) oral medication, (1) insulin), time since diagnosis in years (continuous), complications (dichotomous; (0) no complications, (1) one or more complications), comorbidities (dichotomous; (0) no comorbidities, (1) one or more comorbidities), HbA1c values (continuous), and diabetes-related distress (continuous). All variables, except for diabetes-related distress, were retrieved from patients’ records. In case we had no access to the record, this information was added from self-report. Diabetes-related distress was measured by The Problem Areas In Diabetes (PAID) [44,45]. The internal consistency of the PAID was high in the current study (α = 0.95).

### Sample size calculation

The sample size calculation was based on the primary aim of the original RCT to test the effectiveness of CBT and MBCT in comparison to a waiting list control conditions. Assuming a statistical power of 0.8 and an alpha of 0.05, 42 participants were required in each group enabling us to detect differences with an effect size of 0.6 [46]. As the current study is a secondary analysis of the original RCT, results are regarded as exploratory.

### Data analyses

Internal consistency of the questionnaires was tested by calculating Cronbach’s alpha. Significant preexisting differences between the CBT and MBCT condition were examined by means of t-tests and chi square tests. Analyses were conducted according to the intention-to-treat approach. Missing values were imputed by means of multiple imputations using the Fully Conditional Specification (FCS) method computing five imputed datasets. All analyses were performed using SPSS 23 (see S1 Table for the dataset).

Along with other assumptions, data were checked on multicollinearity. The Variance Inflation Factor (VIF) together with value of tolerance were inspected for continuous variables and the phi-coefficient for categorical variables. A VIF above 10 and a phi above 0.3 are considered as indicators of multicollinearity.

To investigate which patient characteristics predicted and moderated treatment outcome, hierarchical linear regression analyses were conducted including post-treatment BDI-II scores (severity of depressive symptoms) as the dependent variable.

We analyzed potential predictors stepwise. First, we evaluated potential prescriptive predictors (moderators) of CBT and MBCT. For each domain, a model was generated including pre-treatment BDI-II, condition ((1) CBT or (0) MBCT), possible confounding factors, and potential predictors in the first block. The condition x potential predictor interactions were entered in the second block. Next, all significant (alpha 0.05) predictor effects from the different domains (both main and interaction effects) were analyzed in one model to account for potential confounding effects of other predictors.

In case of a significant interaction effect, separate regression analyses were performed for each condition, including post-treatment BDI-II as dependent variable, pre-treatment BDI-II scores, possible confounding factors, and the predictor.

Second, we investigated significant main effects in the final model as prognostic predictors. Significant effects were examined separately for CBT and MBCT to assess whether the prognostic predictor was found irrespective of or in relation to treatment.

We chose to investigate effects in a multivariate approach by selecting different domains in order to reduce inflations of probability of making Type 1 error. To decrease the risk of over-compensation, an alpha of 0.05 was considered statistically significant.

Between-group effect sizes were calculated using Cohen d, with values ranging from 0.2 to 0.5 indicating small effects, values from 0.5 to 0.8 indicating moderate effects, and values > 0.8 indicating large effects [47]. Moderating effects were illustrated in plots of predicted values using Microsoft PowerPoint 2010.

In order to examine whether the effects remained present over time, all analyses were repeated with the BDI-II scores at 9-months follow-up as the dependent variable.

## Results

### Patient characteristics

The sample consisted of 91 Caucasian participants. Patient characteristics are presented in Table 1. Overall, participants had a mean age of 53, 50% was female, and most patients were in a relationship. There was chance baseline imbalance between groups on several variables. Participants randomized to the CBT condition were older, more likely to have one or more comorbidities, and scored lower on neuroticism as compared to the MBCT condition. These variables were controlled for in relevant analyses by including them as a main effect.

**Table 1:** Patient characteristics.

	Total (n = 91)	CBT (n = 45)	MBCT (n = 46)
Age (years) M (SD)*	53.2 (11.9)	56.1 (10.5)	50.4 (12.7)
**Gender n (%)**			
Male	45 (50%)	21 (47%)	24 (52%)
Female	46 (50%)	24 (53%)	22 (48%)
**Education n (%)**			
Lower levels vocational school	21 (23%)	12 (27%)	9 (19%)
Secondary education/advanced level vocational school	52 (57%)	25 (55%)	27 (59%)
Higher or University education	18 (20%)	8 (18%)	10 (22%)
**Employment n (%)**			
Unemployed	39 (43%)	20 (44%)	19 (41%)
Employed	52 (57%)	25 (56%)	27 (59%)
**Marital status n (%)**			
Not in a relationship	25 (27%)	15 (33%)	10 (22%)
In a relationship	66 (73%)	30 (67%)	36 (78%)
**Type of diabetes n (%)**			
Type I	35 (39%)	13 (29%)	22 (48%)
Type II	56 (61%)	32 (71%)	24 (52%)
**Diabetes treatment n (%)**			
Oral medication only	12 (13%)	7 (16%)	5 (11%)
Insulin	79 (87%)	38 (84%)	41 (89%)
**Time since diagnosis (years) M (SD)**	16.7 (12.0)	16.5 (12.6)	16.8 (11.6)
Diabetes complications n (%)**			
No complications	60 (66%)	27 (60%)	33 (72%)
One or more complications	31 (34%)	18 (40%)	13 (28%)
Comorbidity n (%)*			
No comorbidity	42 (46%)	16 (36%)	26 (57%)
One or more comorbidities	49 (54%)	29 (64%)	20 (43%)
**IFCC HbA1c mmol/mol M (SD)**	63.0 (12.4)	64.3 (14.6)	61.7 (9.8)
**Diabetes-related distress M (SD)**	38.8 (21.3)	39.8 (22.3)	37.7 (21.3)
**Current depression n (%)**			
No major depression	67 (74%)	30 (67%)	37 (80%)
Major depression	24 (26%)	15 (33%)	9 (20%)
**History of depression n (%)**			
No history	50 (55%)	23 (51%)	27 (59%)
History	41 (45%)	22 (49%)	19 (41%)
**History of care n (%)**			
No history	46 (51%)	25 (56%)	21 (46%)
History	45 (49%)	20 (44%)	25 (54%)
Neuroticism M (SD)*	40.8 (6.6)	39.5 (6.1)	42.2 (6.8)

* significant differences between the groups at p < 0.05.

** included diabetes complications are: retinopathy, neuropathy, nephropathy, and diabetic foot.

### Prescriptive predictors of change

Data were assessed on multicollinearity. All VIF scores were below 10, indicating no multicollinearity for continuous variables. Type of diabetes and diabetes treatment and type of diabetes and diabetes comorbidity had a phi-coefficient slightly above 0.3 (0.308 and 0.310). Considering that type of diabetes and insulin are also theoretically closely related constructs, yet type of diabetes and diabetes comorbidity not necessarily, we decided to remove diabetes treatment from the analyses.

Fifteen different putative predictors were investigated in three different domains. In the final model combining all significant effects of the analyses within the three domains, a main effect was found for history of care (b = 4.11, SE = 1.64, p = 0.013) and one interaction effect for condition x education (dummy high levels of education; b = 8.17, SE = 3.54, p = 0.021) at post-treatment. For interpretation of the results, means for depressive symptoms and effect sizes of significant predictors are shown in Table 2.

**Table 2:** Mean depression scores for levels of education and history of care, separately for CBT and MBCT on all measurements.

	Group	T1*	T2	T3	Cohen’s d T1-T2 (95%CI)	Cohen’s d T1-T3 (95%CI)
		M (sd)	M (sd)	M (sd)		
**Education**						
Low levels	CBT	26.4 (10.9)	19.0 (13.0)	20.3 (9.9)	0.62 (-0.2–1.4)	0.59 (-0.2–1.4)
	MBCT	25.9 (4.9)	22.6 (9.2)	23.8 (12.2)	0.45 (-0.5–1.4)	0.22 (-0.7–1.1)
Medium levels	CBT	24.2 (7.0)	14.9 (8.2)	17.6 (10.3)	1.23 (0.6–1.8)	0.75 (0.2–1.3)
	MBCT	24.8 (9.3)	17.8 (11.2)	16.4 (10.4)	0.68 (0.1–1.2)	0.85 (0.3–1.4)
High levels	CBT	23.5 (8.6)	21.7 (12.4)	19.8 (14.1)	0.17 (-0.8–1.1)	0.32 (-0.7–1.3)
	MBCT	21.0 (7.8)	12.4 (11.0)	11.7 (7.1)	0.90 (-0.1–1.8)	1.24 (0.2–2.1)
**History of care**						
No history	CBT	24.4 (7.8)	14.9 (9.7)	18.1 (10.5)	1.09 (0.5–1.7)	0.68 (0.1–1.2)
	MBCT	25.5 (9.4)	15.1 (11.7)	15.5 (12.3)	0.98 (0.3–1.6)	0.91 (0.3–1.5)
History	CBT	25.0 (9.1)	20.1 (11.1)	19.5 (11.3)	0.49 (-0.2–1.1)	0.54 (-0.1–1.2)
	MBCT	23.1 (7.4)	19.6 (10.3)	18.0 (9.6)	0.39 (-0.2–0.9)	0.60 (0.0–1.2)

* Measurements: T1 = pre-treatment, T2 = post-treatment, T3 = 9-months follow-up.

**Fig 2:**
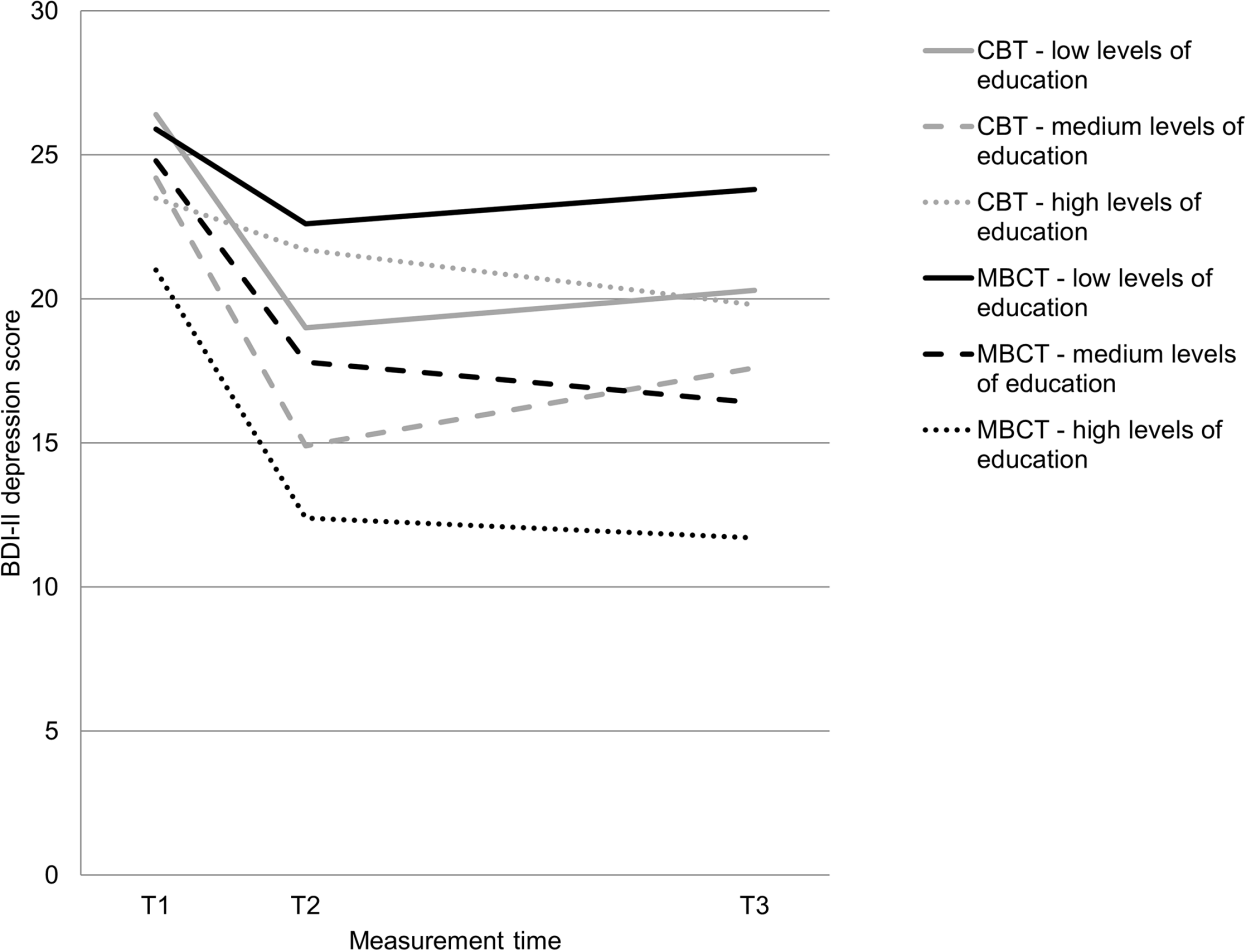
Education as a moderator of depression (BDI-II). CBT = cognitive behavior therapy; MBCT = mindfulness-based cognitive therapy; T1 = pre- treatment, T2 = post-treatment, T3 = at 9-months follow-up.

The interaction effect showed that individuals with high levels of education as compared to individuals with medium or lower levels benefitted less from treatment in the CBT condition than in the MBCT condition (see also Fig 2). Thus, directly after treatment, MBCT might be more advantageous than CBT for individuals with higher levels of educational attainment. Investigating the effect of education on post-treatment depressive symptoms separately for CBT and MBCT revealed that it remained a significant predictor within the CBT condition (b = 7.51, SE = 2.98, p = 0.012), but not within the MBCT condition (b = -1.54, SE = 2.56, p = 0.55). At 9-months follow-up, no significant effects were found, neither main effects, nor interaction effects.

### Prognostic predictors of change

The significant main effect from the final prescriptive model was further investigated to assess whether the effect was generally prognostic and thus irrespective of treatment, or significant only for one treatment. Treatment specific analyses showed that history of care was a significant predictor in both conditions (CBT: b = 4.66, SE = 2. 34, p = 0.047; MBCT: b = 7.13, SE = 1.92, p < 0.001). These results imply that individuals with a history of psychological treatment had less strong treatment effects as compared to individuals who did not receive prior care.

## Discussion

The main purpose of the present study was to investigate whether subgroups of patients with diabetes and comorbid depressive symptoms could be identified that benefitted more from CBT than from MBCT or vice versa. Educational attainment was the only factor that differentially predicted treatment outcomes. The subgroup of individuals with higher educational attainment (relative to individuals with medium or lower levels) reported more favorable outcomes in the MBCT condition than in the CBT condition at post-treatment. This effect was found directly after treatment and did not sustain at 9-months follow-up. For all other demographics, disease-related characteristics, clinical and personality factors, no moderating effects were found. With respect to prognostic predictors (i.e. predictors within treatment conditions), individuals with a history of psychological or psychiatric treatment (relative to those without a history of care) had poorer treatment outcomes in CBT and MBCT. Again, this effect was only evident directly after the intervention and did not remain at 9-months follow-up.

One finding is that level of education was found as a prescriptive predictor of treatment outcome in CBT and MBCT directly after the intervention. Yet, education could not be identified as prognostic predictor within the MCBT condition, but only within the CBT condition. As noted in the introduction, earlier studies that directly compared CBT and MBCT investigated only clinical factors and overlooked other potential prescriptive predictors such as demographics and personality. Previous investigations comparing CBT with Interpersonal Psychotherapy (IPT) or examining CBT and MCBT separately, have neither found that education was a prescriptive nor a prognostic predictor of treatment outcome [26,48,49]. As our findings are inconsistent with findings of earlier studies and as the effect was only temporary, it might be due to chance. Replication of this result with a larger sample size is needed to estimate the magnitude of our finding.

A prognostic predictor irrespective of treatment condition was history of psychological treatment. Patients who received psychological or psychiatric therapy prior to the study had on average lower improvements in depression scores after treatment. Again, when investigating long-term effectiveness, history of treatment did not have an effect. To the best of our knowledge, no existing study investigated the predictive effects of history of psychological treatment. Strikingly, history of recurrent episodes of depression did not predict response to CBT or MBCT in the current study. Also, history of treatment was not related to history of depression, therefore it is unclear whether previous treatment was related to a depressive episode or other psychological problems. Accordingly, more research is needed to assess the predictive influence of history of psychological treatment and care on the effectiveness of CBT and MCBT.

An important observation from this study is that only one prognostic predictor of treatment outcome could be identified, which, in addition, was solely significant immediately after treatment and not at 9-months follow-up. Consistent with previous research [30,33], most demographics, clinical characteristics, or diabetes related characteristics did not moderate or predict outcome. Thus, there was no evidence that either CBT or MBCT was more effective for individuals with certain age, gender, marital status, type of diabetes, or diabetes-related distress. In addition, current results add to the body of research proposing that presence of major depression and history of depressive episode neither moderate nor predict outcome [34,50–53]. This finding seems to be in contradiction with previous MBCT literature stating that MBCT is only effective in preventing recurrence of depression in patients with three or more previous depressive episodes [36,37] and in line with a study finding that MCBT was also effective for individuals with one or two prior episodes [54]. Yet, those studies focused on recurrently depressed patients in remission, whereas the current study investigated patients with current depressive symptoms.

Another main finding was that, in contradiction with our hypothesis, neuroticism neither moderated nor predicted the effects of CBT and MBCT. Whereas some research suggests a tendency to attribute high levels of neuroticism to disadvantageous CBT outcomes [30–32] and beneficial MBCT outcomes [38], other studies cannot validate this trend [30,35]. Those studies were, however, not conducted in patients with diabetes. In a recent study in patients with diabetes, negative affect, a concept closely related to neuroticism, was not identified as a predictor [35]. It is therefore not surprising that our study found no effect.

A plausible explanation of current findings is that CBT and MBCT are accessible interventions that are effective for a broad population of patients with diabetes, including for example old and young people and individuals with severe or mild symptoms. Nevertheless, it could be possible that other factors that are less easy to assess for clinicians, such as attachment style, or coping style would differentially predict treatment outcome. Given the scarcity of previous research comparing CBT and MBCT, more studies are needed to explore other potential prognostic factors and draw firm conclusions.

As CBT and MBCT are general, not diabetes specific interventions to improve depressive symptoms, we believe that current findings can be generalized to other populations. No diabetes specific characteristic was identified as a prognostic predictor, which might indicate that disease related characteristics are less important in choosing the most effective treatment for a given person.

The main limitations of the randomized trial (as also discussed in our earlier paper) include an underpowered sample size and relatively high attrition rates [16]. The current study is therefore likely to be underpowered and analyses are seen as exploratory. Another limitation of this study is that history depression and treatment were assessed by means of self-report, which has possibly resulted in bias.

## Conclusion

The current study is the first to directly compare a range of prognostic and prescriptive predictors for CBT and MBCT for the treatment of depressive symptoms in patients with diabetes. As both CBT and MBCT are effective interventions for reducing depressive symptoms, identifying for whom which treatment may be the most beneficial has implications for clinical decision making. Despite examining a large number of potential prognostic and prescriptive predictors, only few variables influenced treatment response and the effects did not sustain in the long term. These results suggest that patient characteristics only make a modest contribution to treatment outcome and that both treatments are effective for individuals with different characteristics. Future research could consider the contribution of other plausible prognostic factors, or map differences in the therapeutic process, to further advance our understanding of differential response to psychotherapy.

## Supporting information

S1 File Study protocol approved by the Medical Research Ethics committee of the University Medical Center Groningen. (PDF)

S2 File CONSORT statement. (PDF)

S1 Table Dataset what works best for whom. (SAV)
