# Supplementary material for: What works best for whom? Cognitive Behavior Therapy and Mindfulness-Based Cognitive Therapy for depressive symptoms in patients with diabetes
Source: PLoS One. 2017 Jun 29;12(6):e0179941. doi: 10.1371/journal.pone.0179941 (PMC5491069; doi:10.1371/journal.pone.0179941)
Supplement: S1 File — (PDF) [file pone.0179941.s001.pdf]

Mindfulness-Based Cognitive Therapy (MBCT) and Cognitive Behavioral Therapy (CBT) for Depression in Patients with Diabetes: a randomized controlled trial

PROTOCOL TITLE 'Mindfulness-Based Cognitive Therapy (MBCT) and Cognitive Behavioral Therapy (CBT) for Depression in Patients with Diabetes'

Protocol ID	MBCT-CBT-DEPR-D
Short title	MBCT and CBT for depression in patients with diabetes
Version	5
Date	August 2011
Coordinating investigator/project leader	Prof. dr. R. Sanderman<br>University Medical Center Groningen<br>Department of Health Psychology<br>A. Deusinglaan 1<br>9713 AV Groningen<br>-31-50-3636275<br>R.Sanderman@med.umcg.nl
Principal investigator(s) (in Dutch: hoofdonderzoeker/uitvoerder)	MSc. K. A. Tovote and MSc. E. Snippe<br>University Medical Center Groningen<br>Department of Health Psychology<br>A. Deusinglaan 1<br>9713 AV Groningen<br>+31-50-3632955 / +31 50 3632945<br>K.A.Tovote@med.umcg.nl<br>E.Snippe@med.umcg.nl
Project group	Prof. dr. P. Emmelkamp, University of Amsterdam<br>Prof. dr. T. Links, University Medical Center Groningen<br>Prof. dr. I.P. Kema, University Medical Center Groningen<br>dr. J. Fleer, University Medical Center Groningen<br>dr. M.J. Schroevers, University Medical Center Groningen
Sponsor (in Dutch: verrichter/opdrachtgever)	UMCG
Independent physician	dr. A.N.A van der Horst-Schrivers<br>University Medical Center Groningen<br>Department of Endocrinology<br>PO Box 30001<br>9700 RB Groningen<br>+31-50-3616161<br>A.N.A.van.der.Horst@int.umcg.nl

PROTOCOL SIGNATURE SHEET

Name	Signature	Date
Prof. dr. R. Sanderman		
MSc. K. A. Tovote		
MSc. E. Snippe		

SUMMARY

Rationale: Depression is a common co-morbidity of diabetes, negatively affecting physical performance, glycemic control, adherence to medication, and dietary, and exercise recommendations. Modalities of psychotherapy like cognitive behavioral therapy (CBT) and mindfulness-based cognitive therapy (MBCT) can reduce depressive symptoms in patients with medical conditions. However, proper designed randomized trials assessing and comparing effectiveness of these psychological interventions are rare.

Objective: This longitudinal study aims to investigate the effectiveness of CBT and MBCT in reducing depressive symptoms in diabetes patients. Furthermore, potential moderators and mediators of treatment effect will be explored, as well as the role of treatment integrity and therapeutic relationship in CBT and MBCT.

Study design: In a randomized controlled trial, patient will be assigned to one of three conditions: CBT, MBCT, or a wait-list control group. Study parameters will be assessed pre treatment, after the second and after the fifth treatment session, post treatment, 6 months and 12 months follow-up from baseline. Questionnaires and interviews will be used to determine the study parameters. In addition, video-recordings of therapy sessions will be analyzed to find out whether the treatment was carried out as designed (treatment integrity).

Intervention: The intervention consists of 8 weekly individual sessions of CBT or MBCT. Each session will be administered individually and will last for 45 minutes.

Main study parameters: The primary outcome is severity of depressive symptoms as assessed with the Beck Depression Inventory-II and the Toronto Hamilton Depression Rating Scale. Secondary outcomes are well-being, anxiety, and diabetes related distress.

Nature and extent of the burden and risks associated with participation, benefit and group relatedness: The burden for patients consists of completing a written questionnaire (at six points in time for the intervention condition and seven points in time for the wait-list control condition, with time-costs per assessment varying from 5 to 45 minutes) and a short structured interview (5 minutes in the intervention conditions and 2x5 minutes in the wait-list control condition). Furthermore, patients receive treatment in which techniques are used that have proven to be effective in the treatment depression and prevention of recurrence of depression (time costs: 8 weekly sessions of 45 minutes each and approximately 30 minutes of daily homework). We know of no negative effects of these treatments. Therefore, we consider the risks of this study to be low, as patients are primarily asked to invest time and effort.

INTRODUCTION AND RATIONALE

Link between diabetes and depression

The prevalence of diabetes mellitus increases. Due to demographic aging, urbanization, and increasing occurrence of unhealthy lifestyle the number of individuals with diabetes will more than double during the first third of the 21st century (King, Aubert, & Herman, 1998; Wild, Roglic, Green, Sicree, & King, 2004). Depression is a common comorbidity of diabetes. Several studies conclude that diabetes increases or even doubles the risk of developing depression compared to people without a chronic disease. Rates range from 11% to 20% (Ali et al., 2006; Anderson et al., 2001; Fisher et al., 2007). In the population without chronic physical disease, prevalence of depression is 3.2% (Moussavi et al., 2007). Growing evidence from clinical studies indicates that coexistence of depression and diabetes has several implications for patients' physical and mental functioning. It is associated with lower adherence to medication, dietary, and exercise recommendations and poorer physical performance (Ciechanowski, Katon, & Russo, 2000; Ciechanowski, Katon, Russo, & Hirsch, 2003) and also with hyperglycemia (Lustman et al., 2000). To conclude, the presence of comorbid depression in diabetes is associated with diabetes complications leading to morbidity, mortality, and increased healthcare costs (Egede & Ellis, 2010).

Psychological treatment of depression in diabetes patients

Given the high comorbidity of depression in diabetes and its potential negative impact, effective treatment is required to improve depression on the one hand and diabetes self-management ability and adherence on the other hand. Both pharmacologic and psychotherapeutic treatment approaches yield promising results in treating depression in diabetic patients (Lustman & Clouse, 2002). Taken this into account, psychotherapy can be regarded as treatment of choice since nonpharmacologic depression treatments are preferred by the majority of patients (Dwight-Johnson, Sherbourne, Liao, & Wells, 2000). Additionally, one of the important advantages of psychological over pharmacological approach to treat depression is that most psychological therapies involve providing patients with tools which can enable them to cope with future symptoms of depression, and thereby attempt to achieve lower relapse rates of this highly recurrent disorder.

In a randomized controlled trial, Lustman, Griffith, Freeland, Kissel, & Clouse (1998) studied the additive value of cognitive behavioral therapy (CBT) to a diabetes education program in treating major depression in diabetes patients (n=51). The authors found that addition of CBT is more effective in treating major depression than an education program alone. Likewise, Georgiades et al. (2007) found that CBT intervention to treat depression in diabetic patients lead to reduction in depressive symptoms over a 12-month period (n=90).

Furthermore, van der Ven et al. (2005) and Amsberg et al. (2009) examined the impact of CBT on diabetes self-efficacy and well-being in non-depressed diabetes patients with prolonged poor glycemic control in randomized controlled trials with sample sizes of respectively 107 and 74. In both studies, cognitive behavioral group training was found to be effective in reducing diabetes-related distress, depressive symptoms and increasing diabetes self-efficacy. To conclude, previous research found that

CBT is an effective intervention for treating depressive symptoms in diabetes patients. In addition, it has a positive effect on diabetes self-efficacy.

Cognitive Behavioral Therapy (CBT) versus Mindfulness-Based Cognitive Therapy (MBCT)

Recently, mindfulness-based therapy has been gaining popularity in both research and clinical practice. Mindfulness refers to being aware of the present moment by paying attention on purpose and without judgment (Kabat-Zinn, 2003). Mindfulness-based cognitive therapy (MBCT) has been addressed as a third wave psychological treatment elaborating on CBT (Hofmann & Asmundson 2008). Mindfulness-based interventions have been found to be effective in reducing emotional and psychological components associated with chronic disease (Shigaki, Glass, & Schopp, 2006). In non-depressed diabetes patients, a one-day workshop focusing on acceptance and mindfulness improved coping strategies and self-management significantly more than a traditional diabetes education workshop (Gregg, Callaghan, Hayes, & Glenn-Lawson, 2007).

Although research about clinical applications of mindfulness is growing in the last decades, it is still novel compared to other types of psychotherapy, such as CBT. Especially, studies about the long term effects are rare. To date, no randomized controlled trials of MBCT have been carried out in depressed patients with diabetes. Furthermore, little research has been done to compare CBT and MBCT directly as two active treatments (see for example Piet et al., 2010).

The present study will undertake such a comparison, both to study the immediate and long-term effects of the two treatments compared to a wait-list control group and to seek insight in potential moderators of treatment effects and mediators underlying the effects. Additionally, the role of treatment integrity and therapeutic relationship in treatment effects will be examined.

Moderators

In order to examine for whom CBT and MBCT are (not) effective, several moderators will be examined. When the relationship between two variables depends on a third variable, this third variable is considered a moderator. Moderating variables influence the direction and / or strength of the relationship between two variables (Baron & Kenny, 1986). Results from previous studies suggest that certain patient characteristics function as moderators of the relation between type of treatment and treatment effect (Lustman, Freedland, Griffith, & Clouse, 1998).

One such factor is history of recurrent depression. The present study will examine history of depression as a possible moderator of treatment effect. Based on previous findings (Zautra et al., 2008) we expect a greater effect of MBCT in patients with past episode of depression. Compliance with self-monitoring of blood glucose can be imagined as another moderator (Lustmann et al., 1998). Lustmann et al. (1998) found that CBT intervention was overall effective in reducing depressive symptoms, but that patients scoring lower on compliance with self-monitoring of blood glucose (SMBG) and with presence of diabetes complications achieved less remission of depressive symptoms than did patients with higher compliance with SMBG and no diabetes complications. Therefore we expect that patients with elevated HbA_{1c} levels (e.g., ≥8%) profit less from treatment compared to patients with normal HbA_{1c} values.

Furthermore, personality traits such as neuroticism, may moderate the relationship between type of treatment and response to treatment. Bagby et al. (2008) found that individuals who scored high on neuroticism had less benefit from CBT compared to treatment with an antidepressant, possibly because those with high scores on neuroticism may be emotionally too dysregulated to be able to effectively use cognitive strategies required for successful CBT. Taken this into account, the present study will examine whether people scoring high on this trait respond better to MBCT than to CBT because they learn to accept content of their thoughts without trying to change them.

Additionally, attachment styles, which reflect bonds between individuals, will be considered as moderator of treatment outcome. Cordon, Brown, and Gibson (2009) found that insecurely attached individuals benefit most from Mindfulness-Based Stress Reduction (MBSR) program compared to securely attached individuals. In a different randomized controlled trial, it was found that avoidant attached patients responded better to CBT as compared to Interpersonal Psychotherapy (IPT) (McBride, Atkinson, Quilty, & Bagby, 2006). Attachment styles a moderator for treatment outcome in CBT and MBCT have not been studied yet. Therefore this study aims at exploring the moderating function of attachment.

Finally, physiological measures could influence effect of treatment. Type 2 diabetes and obesity are linked with a low-grade inflammation state, leading to an elevation of the immunomodulatory enzyme indoleamine 2,3-dioxygenase (IDO). IDO degrades the essential amino acid tryptophan to kynurenine causing depletion of tryptophan (Brandacher, Hoeller, Fuchs, & Weiss, 2007). These changes in tryptophan plasma levels lead to a reduction in serotonin production and cause mood disturbances (Russo, Kema, Bosker, Haavik, & Korf, 2009). Functioning as a natural antidepressant (Thomson, Rankin, Ashcroft, Yates, McQueen, Cummings, 1982), tryptophan could influence treatment outcome. In addition, dysregulation of the neuroendocrine system (catechol-O-methyl transferase [COMT] and monoaminse oxidase A [MAOA]) is associated with depression (Jabbi et al., 2007). In the current study, participants with high levels of tryptophan and a regulated level of the neuroendocrine system are expected to benefit more from treatment than participants with low levels of tryptophan.

Mediators

To examine why CBT and MBCT are (not) effective, several mediators will be examined. Mediators are variables through which one variable influences another variable (Baron & Kenny, 1986). Mediators explain the mechanisms underlying the relationship between two variables. For example, CBT can enhance certain skills (mediators) by which depressive symptoms are reduced. To get more insight in the patterns of change in CBT and MBCT, it will be studied whether treatment outcomes are mediated by different mediators in CBT and MBCT.

Behavioral activation and challenging and reducing negative automatic thoughts are major components of CBT which are not included in MBCT. Accordingly, hypothesized mediators of treatment effects of CBT are behavioral activation and reappraisal. Training of attention, acting with awareness, acceptance, and non-judging are components of MBCT which are not included in CBT. Therefore, the hypothesized mediators of treatment effects of MBCT are attentional control, acting with awareness, non-judgmental attitude and self-compassion. Furthermore, it is expected that CBT

and MBCT will have several mediating variables in common, as MBCT is founded on the principles of CBT. The hypothesized common mediators of CBT and MBCT are positive re-interpretation, positive refocusing, rumination, seeking distraction and overidentifitcation.

Treatment integrity and the therapeutic relationship

Firm conclusions about differential efficacy of treatments can only be drawn when information is available on whether the treatment was carried out as designed, known as treatment integrity (Perepletchikova, 2009). Research on treatment integrity contributes to explaining (non)significant results, increases internal validity, and is useful for study replication and generalization (Moncher & Prinz, 1991; Borrelli et al., 2005; Resnick et al., 2005). The three following aspects of treatment integrity will be taken into account in the present study: treatment adherence, treatment differentiation and treatment enactment. Treatment adherence refers to the extent to which the therapist adheres to procedures described in the protocol and avoids non prescribed procedures (Perepletchikova et al., 2009). Whether the treatments differ in the intended manner is referred to as treatment differentiation (Moncher & Prinz, 1991). Treatment enactment is the degree to which patients apply skills related to the intervention in their daily lives (Burgio, 2001). Homework compliance is assessed as a measure of treatment enactment in the present study.

The therapeutic relationship is one of the main predictors of outcome in a broad range of psychological treatments (Horvath & Symonds, 1991). The therapeutic relationship has been shown to be related to positive treatment outcome in CBT (Keijsers et al., 2000). It is suggested that being more mindful as a psychotherapist enhances the development of a therapeutic relationship and promotes treatment outcome (Bruce, 2010; Grepmair, 2007). However, the specific role of the therapeutic relationship in the process of change in MBCT has not been studied yet. Moreover, many studies do not consider the important interactions between treatment techniques and the therapeutic relationship (Huppert, 2006). Therefore, we will study (1) the development of the therapeutic relationship in CBT and MBCT (2) the interrelationship between the therapeutic relationship, treatment adherence and homework compliance (3) to what extent these treatment factors predict changes in hypothesized CBT and MBCT mediators and treatment outcomes.

OBJECTIVES

Primary Objective:

- To assess immediate and long-term effects of Cognitive Behavioral Therapy (CBT) and Mindfulness-based Cognitive Therapy (MBCT) in reducing depression in diabetes patients
  Hypothesis: Regarding the immediate effects, we expect that both CBT and MBCT are more effective than a wait-list control condition in reducing depressive symptoms in diabetes patients.

Secondary Objectives:

- To examine factors that mediate treatment effects of CBT and MBCT
- To examine factors that moderate treatment effects of CBT and MBCT
- To examine the role of treatment integrity and therapeutic relationship in treatment effects of CBT and MBCT

STUDY DESIGN

A multicenter randomized controlled trial with a longitudinal design will be conducted. Eligible patients who want to participate will be randomly assigned to CBT, MBCT, or a wait-list control group. Using a wait-list condition will allow us to partially control for the possibility that in some patients natural recovery from depression may occur. Patients in the CBT and MBCT condition will be assessed before start of treatment (T1), twice during treatment (T2 and T3), immediately after ending of treatment (T4), at a 6-month follow-up from baseline (i.e., 3 months after ending of treatment; T5), and at a 12-month follow-up from baseline (i.e., 9 months after ending of treatment; T6). Patients in the wait-list control condition will undertake assessments at similar time points, except that they will undertake the baseline assessment (T1) twice, once before start of treatment in the CBT/MBCT conditions ($T1_a$) and once at the end of the three-months waiting period ($T1_b$).

STUDY POPULATION

Population (base)

Study participants are diabetes patients who suffer from at least mild symptoms of depression as assessed with the Beck Depression Inventory (BDI-II). The patients will be recruited at the University Medical Center Groningen (UMCG). Furthermore, we plan to ask other hospitals in the North of the Netherlands to cooperate (the Martini Hospital in Groningen, the Wilhelmina Hospital in Assen, and the Refaja Hospital in Stadskanaal).

Inclusion criteria

- Diabetes mellitus Type 1 or 2 for at least three months prior to inclusion
- Written informed consent
- Age ≥ 18 and ≤ 70
- Depressive symptoms as assessed by BDI-II score ≥ 14 (cut-off score indicating the presence of at least mild symptoms of depression)

Exclusion criteria

- Not being able to read and write Dutch
- Severe (psychiatric) co-morbidity
- Acute suicidal ideations or behavior
- Pregnancy
- Currently receiving alternative psychological treatment for depression

Using an antidepressant during participation in the present study is allowed, on condition that a patient has been on stable medication regimen for at least two months prior to inclusion in the study, and that no new treatment with an antidepressant is initiated during the course of the study. By restricting the antidepressant use this way we attempt to ensure that changes in patients’ mood are due to their participation in the study conditions rather than in due to introduction or modification of dosage of an

antidepressant medication. In case of an apparent need or wish of the patient to start a pharmacologic treatment for depression during the course of the study, the patient may complete CBT or MBCT treatment but his or her data will be excluded from the analyses.

Sample size calculation

The sample size calculation is based on differences in depressive symptoms post treatment between the wait-list control group and one of the psychological intervention groups. A 5 point difference on the Beck Depression Inventory-II (assuming a standard deviation of 8 points) between the wait-list control group and one of the intervention groups is considered a relevant difference. We aim to include at least 42 patients per group (126 patients in total). With 42 patients per group, a power of 80%, an alpha of 0.05 (tested two-sided) we will be able to detect an effect size of 0.6 according to Cohen (1992).

TREATMENT OF SUBJECTS

The MBCT treatment protocol is based on the protocol as developed by Segal, Teasdale, and Williams (2002). Key themes include experimental learning and the development of an open and acceptant mode of negative feelings, and body sensations, allowing them to be "nipped in the bud" at an early stage. Formulation of specific prevention strategies is included in the later stages of treatment. Originally, MBCT is given as a group treatment, but in 2009 an individualized version of this protocol was developed at the department of Health Psychology by a certified MBCT-trainer and GZ-psychologist (Dr. M.J. Schroevers, department of Health Psychology). This protocol has recently been tested in a pilot study among diabetes patients (see METC protocol 'Psychologische behandeling bij diabetes burnout', Fleer et al. 2008), and will only need minor adjustments to be of use in this study.

The pilot study yielded promising results. Univariate repeated measures analyses (ANOVAs) showed that MBCT is effective in treating depression. Participants in the treatment condition showed a reduction of depressive symptoms (assessed with the CES-D) and diabetes related distress (assessed with the PAID), whereas patients in the waitlist condition showed no reduction of symptoms over time [CES-D: $F(1,18) = 15.28$, $p<0.001$; PAID: $F(1,19) = 15.28$, $p<0.001$]. At 6-months follow-up, effects in treatment group stayed constant. There was no significant difference in scores on CES-D and PAID directly after treatment and at follow-up [CES-D: $F(1,9) = 2.22$, $p=0.17$; PAID: $F(1,9) = 1.83$, $p=0.21$].

The training will be given by psychologists under supervision. The treatment consists of eight 45-minute MBCT sessions.

Session 1 ("Automatic pilot")

- Greeting, shortly explain the purpose of the training
- Bodyscan + evaluation
- Raisin exercise + evaluation
- Providing homework assignments + giving a work book

Session 2 (“Dealing with barriers”)

- Bodyscan + evaluation
- Discussion of homework
- Breathing exercise + evaluation
- Providing homework assignments

Session 3 (“Mindfulness of the breath”)

- Breathing exercise + evaluation
- Discussion of homework
- Mindful Yoga + evaluation
- 3-minute breathing space
- Providing homework assignments

Session 4 (“Staying present”)

- Meditation exercise (attention from breath, body, sound, thoughts) + evaluation
- Discussion of homework
- Recognition of signals of depression relapse
- Mindful Yoga
- Providing homework assignments

Session 5 (“Allowing and letting be”)

- Mediation exercise (attention form breath, body, sound, thoughts) + evaluation
- Discussion of homework
- Recognition of signals of depression relapse – sequel
- Walking meditation
- Providing homework assignments

Session 6 (“Thoughts are not facts”)

- Mediation exercise (attention form breath, body, sound, thoughts) + evaluation
- Discussion of homework
- Thought exercise
- 3-minute breathing space and coping with negative thoughts and feelings
- Providing homework assignments

Session 7 (“How can I best take care of myself”)

- Mediation exercise (attention form breath, body, sound, thoughts) + evaluation
- Discussion of homework
- Exercise relation activities and mood
- Providing homework assignments

Session 8 (“Using what is learned to deal with future moods”)
- Bodyscan + evaluation
- Discussion of homework
- Evaluation of the training

The CBT treatment protocol is based on the CBT protocol for depressed patients by Boelens and Bloedjes (2004). The first part of the treatment is devoted to behavioral components of CBT, such as planning and undertaking of (pleasant or functional) activities. The second part of the treatment focuses on dysfunctional thinking patterns, allowing patients to recognize, challenge and adjust their negative automatic thoughts. The training will be given by psychologists under supervision. The treatment consists of eight 45-minute CBT sessions.

Session 1
- Acquaintance; elaboration on questions of patient which were not clearly addressed during intake
- Explaining treatment rationale
- Instructing patient how to use the activity form (homework in workbook)
- Providing homework assignments

Session 2
- Handling possible questions / remarks on treatment rationale
- Discussion of homework
- Discussion of registration and planning of activities
- Stressing the gradual assignment of activities
- Providing homework assignments

Session 3
- Discussion of homework
- Registration and planning of activities (sequel)
- Providing homework assignments

Session 4
- Discussion of homework
- Explaining the cognitive rationale
- Giving instructions on the use of the diary form
- Providing homework assignments

Session 5
- Answering questions on the cognitive rationale (if applicable)
- Discussion of homework

- Identifying automatic thoughts and fallacies in thinking
- Providing homework assignments

Session 6
- Discussion of homework
- Challenging automatic thoughts
- Formulating rational thoughts
- Scoring and re-scoring of thought credibility
- Providing homework assignments

Session 7
- Discussion of homework
- Challenging automatic thoughts (sequel)
- Formulating rational thoughts (sequel)
- Providing homework assignments

Session 8
- Discussion of homework
- Evaluation and termination of the treatment

METHODS

Main study parameters / endpoints

The primary outcome is severity of depressive symptoms as assessed with the Beck Depression Inventory-II (BDI-II) and the Toronto Hamilton Depression Rating Scale (HAM-D_7). Secondary outcomes are diabetes related distress measured by the Problem Areas in Diabetes scale (PAID), generalized anxiety measured by the GAD-7 and wellbeing measured by the WHO-5.

Assessment of patients

Assessments will take place before treatment (T1; this assessment will be repeated in the wait-list control condition after ending of the waiting period), after the second treatment session (T2), after five treatment sessions (T3), immediately after treatment (T4), 3 months after treatment (T5) and 9 months after treatment (T6). The two measurements during the course of treatment will be used in order to assess the development of therapeutic relationship (T2-T3) and the process of change in mediator and outcome variables (T3). Table 1 presents an overview of which measures will be assessed on which time-point. Assessments at T1 will take patients approximately 45 minutes. Assessments at T2 will take around 5 minutes. Assessments at T3 will take about 35 minutes. Assessments at T4 will take approximately 45 minutes. Assessments at T5 and T6 will take around 30 minutes.

Additionally, severity of depression will be assessed with the Toronto Hamilton Depression Rating Scale (HAM-D_7). This interview is based on 7 items and takes approximately 5 minutes. It is based on the original 17-item Hamilton Depression Rating Scale (Hamilton, 1960) which is widely used to

supplement self-report depression measurement in clinical research. The HAM-D_7 will be administered to all patients during the intake, to the wait-list control group directly before treatment (after the ending of the waiting period) and after the completion of treatment, and to the intervention groups directly after the completion of treatment.

Measures

For an overview of the instruments used to assess primary outcome, secondary outcomes, mediators, and moderators see Table 1.

Table 1

Instruments to assess primary outcome, secondary outcomes, mediators, and moderators

Measure	Explanation	Assessment	No. of Items
Beck Depression Inventory-II (BDI-II) (Beck, 1996)	Depressive symptoms	T1, T3, T4, T5, T6	21
GAD-7 (Spitzer, 2006)	Generalized anxiety	T1, T4, T5, T6	7
WHO-5 (World Health Organization, 1998)	Well-being	T1, T4, T5, T6	5
The Problem Areas in Diabetes scale (PAID) (Polonsky, 1995)	Diabetes-related distress	T1, T4, T5, T6	20
The experiences in close relationship scale short form (ECR-S) (Wei, 2007)	Attachment style	T1, T4	12
NEO- Five Factor Inventory (NEO-FFI) (Hoekstra, 1996)	Neuroticism: negative affect and self-reproach	T1	12
2 subscales of the Cognitive Emotion Regulation Questionnaire (CERQ) (Garnefski, 2007)	Positive re-interpretation and positive refocusing	T1, T3, T4, T5, T6	8
2 subscales of the Thought Control Questionnaire (TCQ) (Wells, 1994)	Reappraisal and distraction	T1, T3, T4, T5, T6	12
1 subscale of the Behavioral Activation for Depression Scale (BADS) (Raes, 2010)	Activation	T1, T3, T4, T5, T6	7
2 subscales of the Five Facet Mindfulness Questionnaire (FFMQ) (Baer, 2008)	Non-judgmental attitude and act with awareness	T1, T3, T4, T5, T6	16
2 subscales of the Self Compassion Scale (SCS) (Neff, 2003)	Self-kindness and overidentification	T1, T3, T4, T5, T6	9
Self-Regulation Scale (Schwarzer, 1999)	Attention control	T1, T3, T4	10
1 subscale of the Rumination-Reflection	Rumination	T1, T3, T4, T5, T6	12

Questionnaire (RRQ) (Trapnell, 1999)			
Working Alliance Inventory (WAI-12) (Vervaeke, 1993)	Patients’ and therapists’ Working alliance	T2, T3, T4	12
18-item Rapport Questionnaire (Bernieri, 2005)	Rapport between patient and therapist	T2, T3, T4	18

Other assessments

Next to self-report questionnaire and interview assessments, several measures will be assessed in other ways. Co-morbidity, severity of the disease, Body Mass Index, medication use, hospitalizations, and HbA_{1c} levels will be retrieved from patients’ records if they give written informed consent. The Working Alliance Inventory (WAI-12; Vervaeke, 1993) and the 18-item Rapport Questionnaire (Bernieri, 2005) will be filled out by the therapist at T2, T3 and T4 as well. Also, the videotaped therapy sessions will be rated by two independent raters. These raters will use session registration lists developed in a pilot study to rate treatment adherence and treatment differentiation (Bouwkamp, 2009). The raters will rate the therapeutic relationship by using the WAI-12 (Vervaeke, 1990).

Furthermore, patients’ blood sample will be used to investigate degradation of tryptophan, catechol-O-methyl transferase (COMT), and monoaminse oxidase A (MAOA) at T1 in patients that give written informed consent.

Procedure

The diabetes outpatient clinic of the University Medical Center Groningen (UMCG) aims to provide high quality psychosocial care for diabetes patients. Therefore, the screening for psychosocial problems and the additional intake, which is aimed at developing a better understanding of the patient's symptoms in order to recommend the most appropriate treatment plan, are part of standard care. Patients who have an appointment scheduled with a physician at the diabetes outpatient clinic receive a short screening questionnaire (i.e., BDI-II) and a return envelope one week prior to their appointment. They are asked to complete the questionnaire and hand it in at the secretariat of the outpatient clinic at the day of their appointment. Patients with a BDI-II score above the cut-off will be invited (by telephone) for a 1-hour face-to-face intake with a psychologist at the diabetes outpatient clinic. During the intake the Toronto Hamilton Depression Rating Scale (HAM-D7) will be administered for obtaining additional diagnostic information about the level of depressive symptoms. Besides that, the patients' motivation for receiving psychological treatment will be checked as well as the fulfillment of the inclusion and exclusion criteria (such as presence of suicidal ideation and pregnancy) for the current study. Motivated and eligible patients will then receive brief information on the psychological interventions. Those who are willing to consider participation in the study receive a booklet with information about the study, the first questionnaire (T1), an informed consent form, and a pre-paid return envelope. They are asked to return a completed informed consent form and the questionnaire within two weeks. Patients are also notified that treatment will start between two weeks and three months after their inclusion in the study. Patients who do not fulfill the in- and exclusion criteria, or are not willing to participate in the study, yet do express a need for further help will be referred to other

treatment resources (the Ambulatorium of Medical Psychology in the UMCG or other psychological or psychiatric services) after consulting their primary caregiver.

All patients enrolled in the trial will also be asked to give informed consent for video taping their treatment sessions. All treatment sessions will be taped of patients that provide consent. Patients will be notified that all video tapes will be destroyed after the completion of research analyses. Video taping the treatment sessions is optional for patients and does not affect their participation in the study. We aim to video tape the treatment sessions of 15 patients per treatment group, 30 patients in total. Recordings will be made by two cameras; one directed at the patient, the other directed at the therapist. A pilot study in which diabetes patients were treated with MBCT showed that 60% of diabetes patients agree that their treatment sessions are video taped.

Furthermore, patients will be asked if they allow sampling a maximum of 3 ml extra blood in order to assess other biochemical measures (tryptophan, COMT, and MAOA). As far as possible, the extra blood will be sampled during routine blood sampling, which is used to examine HbA_{1c} levels so that patients do not need to be pricked twice. Taping the treatment sessions, sampling extra blood and analyzing it, and giving informed consent for retrieving data from records is optional and it does not affect participation in the intervention study.

Subsequently, patients who give their written consent for participation in the intervention will be randomly assigned to CBT, MBCT, or a wait-list control group. Patients assigned to the wait-list control group will be randomized for a second time to one of the treatment conditions after the waiting period of about 3 months. Due to response rates from prior research we expect that about 5% of screened patients are eligible and willing to participate in this study. Consequently we need to screen at least 2520 patients to be able to include three groups of 42 patients in this study. Computerized stratified randomization by gender, treatment with an antidepressant, and age will be used. All patients in the intervention condition will receive treatment as soon as possible, generally within two weeks after randomization. Patients in the wait-list control group will receive treatment with a delay of three months, at maximum. Patients will be enrolled continually at any point during the study inclusion period.

Withdrawal of individual subjects

Subjects can leave the study at any time for any reason if they wish to do so without any consequences. The investigator can decide to withdraw a subject from the study for urgent medical reasons. In case of serious lack of compliance the investigators can also decide to stop a patient’s participation in MBCT or CBT.

SAFETY REPORTING

Section 10 WMO event

In accordance to section 10, subsection 1, of the WMO, the investigator will inform the subjects and the reviewing accredited METC if anything occurs, on the basis of which it appears that the disadvantages of participation may be significantly greater than was foreseen in the research proposal. The study will be suspended pending further review by the accredited METC, except insofar

as suspension would jeopardise the subjects' health. The investigator will take care that all subjects are kept informed.

Adverse and serious adverse events

Adverse events are defined as any undesirable experience occurring to a subject during the study, whether or not considered related to the experimental treatment. All adverse events reported spontaneously by the subject or observed by the investiga-tor or his staff will be recorded.
A serious adverse event is any untoward medical occurrence or effect that at any dose:

- results in death;
- is life threatening (at the time of the event);
- requires hospitalization or prolongation of existing inpatients' hospitalization;
- results in persistent or significant disability or incapacity;
- is a congenital anomaly or birth defect;
- is a new event of the trial likely to affect the safety of the subjects, such as an unexpected outcome of an adverse reaction, lack of efficacy of an IMP used for the treatment of a life threatening disease, major safety finding from a newly completed animal study, etc.

All SAEs will be reported through the web portal *ToetsingOnline* to the accredited METC that approved the protocol. SAEs that result in death or are life threatening should be reported expedited. The expedited reporting will occur not later than 7 days after the responsible investigator has first knowledge of the adverse reaction. This is for a preliminary report with another 8 days for completion of the report.

STATISTICAL ANALYSIS

Intention-to-treat analyses will be done. To answer the primary research question, multivariate analyses will be performed, using primary and secondary outcomes as dependent variables. Psychological intervention will be used as independent variable. It will be controlled for several patient characteristics, as gender, disease severity, prior history of depression, use of antidepressant medication, and severity of depressive symptoms at baseline. Repeated measures analyses will be performed to calculate changes in time. To describe the study population, descriptive statistics will be used analyzing data from T1.

A reduction of 5 points on the BDI-II will be considered as clinical relevant improvement. In accordance with previous research (Keers, Groen, Sluiter, Bouma, & Links, 2005), a Number Needed to Treat of 2.0 is considered cost-efficient and clinical relevant. Stated differently, at least half of participants should improve 5 points on the BDI-II.

The videotaped therapy sessions will be rated by two independent raters. These raters will use session registration lists developed in a pilot study to rate treatment adherence and treatment differentiation (Bouwkamp, 2009). The raters will rate the therapeutic relationship by the WAI-12 (Vervaeke, 1990) as well. Repeated measures analyses and time-series analyses will be used to analyse the data on treatment integrity and the therapeutic relationship.

ETHICAL CONSIDERATIONS

Regulation statement

The study will be conducted according to the principles of the Declaration of Helsinki (version 2008) and in accordance with the Medical Research Involving Human Subjects Act (WMO).

Benefits and risks assessment, group relatedness

The burden for patients consists of completing questionnaires (in total 190 minutes) and a short structured interview (5 minutes in the intervention conditions and 2x5 minutes in the wait-list control condition). The benefit for patients consists of receiving psychological treatments in which techniques are used that have proven to be effective in treatment and prevention of depression. Patients will receive eight 45-minute psychological treatment sessions. The patients will also be asked to engage in homework exercises during the course of treatment (about 30 minutes per day). To our knowledge, there are no negative effects of these treatments. We consider the risks of this study to be low, as patients are primarily asked to invest time and effort.

Compensation for injury

As the risks of this study are considered to be null, we have acquired exemption from insurance (ontheffing van verzekeringsplicht).

ADMINISTRATIVE ASPECTS AND PUBLICATION

Handling and storage of data and documents

Data will be collected and stored in a digital way using Global Park software. Data will be handled confidentially and anonymously. Participants will receive a code number, (the number of entry in the study) and a identification list is kept by the principal investigators. Data are only accessible by the principal investigators and the coordinating investigators

Annual progress report

The investigator will submit a summary of the progress of the trial to the accredited METC once a year. Information will be provided on the date of inclusion of the first subject, numbers of subjects included and numbers of subjects that have completed the trial, serious adverse events/ serious adverse reactions, other problems, and amendments.

End of study report

The investigator will notify the accredited METC of the end of the study within a period of 3 months after ending of study. The end of the study is defined as the completion of the last questionnaire.

In case the study is ended prematurely, the investigator will notify the accredited METC, including the reasons for the premature termination.

Within one year after the end of the study, the investigator/sponsor will submit a final study report with the results of the study, including any publications/abstracts of the study, to the accredited METC.

Public disclosure and publication policy

Results of this research will be published in peer-reviewed journals

References

Ali, S., Stone, M. A., Peters, J. L., Davies, M. J., & Khunti, K. (2006). The prevalence of co-morbid depression in adults with type 2 diabetes: A systematic review and meta-analysis. *Diabetic Medicine : A Journal of the British Diabetic Association, 23*, 1165-1173.

Amsberg, S., Anderbro, T., Wredling, R., Lisspers, J., Lins, P. E., Adamson, U., et al. (2009). A cognitive behavior therapy-based intervention among poorly controlled adult type 1 diabetes patients--a randomized controlled trial. *Patient Education and Counseling, 77*, 72-80.

Anderson, R. J., Freedland, K. E., Clouse, R. E., & Lustman, P. J. (2001). The prevalence of comorbid depression in adults with diabetes: A meta-analysis. *Diabetes Care, 24*, 1069-1078.

Baron, R. M., & Kenny, D. A. (1986). The moderator-mediator variable distinction in social psychological research: Conceptual, strategic, and statistical considerations. *Journal of Personality and Social Psychology, 51*, 1173-1182.

Beck, A. T., Steer, R. A., Ball, R., & Ranieri, W. F. (1996). Comparison of Beck Depression Inventories–IA and –II in psychiatric outpatients. *Journal of Personality Assessment, 67*, 588-597.

Bernieri, F. J. (2005). The expression of rapport. In V. Manusov (Ed.), *The sourcebook of nonverbal measures: Going beyond words.* (pp. 347-359). Mahwah, NJ US: Lawrence Erlbaum Associates Publishers.

Borrelli, B., Sepinwall, D., Ernst, D., Bellg, A. J., Czajkowski, S., Breger, R., et al. (2005). A new tool to assess treatment fidelity and evaluation of treatment fidelity across 10 years of health behavior research. *J.Consult Clin.Psychol.,73*, 852-860.

Brandacher, G., Hoeller, E., Fuchs, D., & Weiss, H. G. (2007). Chronic immune activation underlies morbid obesity: Is IDO a key player? *Current Drug Metabolism, 8*, 289-295.

Bruce, N. G., Manber, R., Shapiro, S. L., & Constantino, M. J. (2010). Psychotherapist mindfulness and the psychotherapy process. *Psychotherapy: Theory, Research, Practice, Training, 47*, 83-97.

Ciechanowski, P. S., Katon, W. J., & Russo, J. E. (2000). Depression and diabetes: Impact of depressive symptoms on adherence, function, and costs. *Archives of Internal Medicine, 160*, 3278-3285.

Ciechanowski, P. S., Katon, W. J., Russo, J. E., & Hirsch, I. B. (2003). The relationship of depressive symptoms to symptom reporting, self-care and glucose control in diabetes. *General Hospital Psychiatry, 25*, 246-252.

Cohen, J. (2003). A power primer. In A. E. Kazdin (Ed.), *Methodological issues & strategies in clinical research (3rd ed.).* (pp. 427-436). Washington, DC US: American Psychological Association.

Cordon, S. L., Brown, K. W., & Gibson, P. R. (2009). The role of mindfulness-based stress reduction on perceived stress: Preliminary evidence for the moderating role of attachment style. *Journal of Cognitive Psychotherapy, 23*, 258-269.

Dwight-Johnson, M., Sherbourne, C. D., Liao, D., & Wells, K. B. (2000). Treatment preferences among depressed primary care patients. *Journal of General Internal Medicine, 15*, 527-534.

Egede, L. E., & Ellis, C. (2010). Diabetes and depression: Global perspectives. *Diabetes Research and Clinical Practice*, 87, 302-312.

Fisher, L., Skaff, M. M., Mullan, J. T., Arean, P., Mohr, D., Masharani, U., et al. (2007). Clinical depression versus distress among patients with type 2 diabetes: Not just a question of semantics. *Diabetes Care, 30*, 542-548.

Garnefski, N., & Kraaij, V. (2007). The cognitive emotion regulation questionnaire: Psychometric features and prospective relationships with depression and anxiety in adults. *European Journal of Psychological Assessment, 23*, 141-149.

Georgiades, A., Zucker, N., Friedman, K. E., Mosunic, C. J., Applegate, K., Lane, J. D., et al. (2007). Changes in depressive symptoms and glycemic control in diabetes mellitus. *Psychosomatic Medicine, 69*, 235-241.

Gregg, J. A., Callaghan, G. M., Hayes, S. C., & Glenn-Lawson, J. L. (2007). Improving diabetes self-management through acceptance, mindfulness, and values: A randomized controlled trial. *Journal of Consulting and Clinical Psychology, 75*, 336-343.

Hoekstra H.A., Ormel J., De Fruyt F. De NEO-PI-R/NEO-FFI; Big Five persoonlijkheidsvragenlijsten; Handleiding. [Manual of the Dutch version of the NEO-PR-I/NEO-FFI]. Lisse: Swets and Zeitlinger, 1996.

Hofmann, S. G., & Asmundson, G. J. G. (2008). Acceptance and mindfulness-based therapy: New wave or old hat? Clinical Psychology Review, 28(1), 1-16.

Horvath, A. O., & Symonds, B. D. (1991). Relation between working alliance and outcome in psychotherapy: A meta-analysis. *Journal of Counseling Psychology, 38*, 139-149.

Huppert, J. D., Fabbro, A., & Barlow, D. H. (2006). Evidence-based practice and psychological treatments. In C. D. Goodheart, A. E. Kazdin & R. J. Sternberg (Eds.), *Evidence-based psychotherapy* (pp. 131-152). Washington, D.C.: American Psychological Association.

Jabbi, M., Korf, J., Kema, I. P., Hartman, C., van der Pompe, G., Minderaa, R. B., et al. (2007). Convergent genetic modulation of the endocrine stress response involves polymorphic variations of 5-HTT, COMT and MAOA. *Molecular Psychiatry, 12*, 483-490.

Kabat-Zinn, J. (2003). Mindfulness-based interventions in context: Past, present, and future. *Clinical Psychology: Science and Practice, 10*, 144-156.

Keijsers, G. P. J., Schaap, C. P. D. R., & Hoogduin, C. A. L. (2000). The impact of interpersonal patient and therapist behavior on outcome in cognitive-behavioral therapy: A review of empirical studies. *Behavior Modification, 24*, 264-297.

Keers, J.C., Groen, H., Sluiter, W.J., Bouma, J., Links, T.P., (2005). Cost and benefits of a multidisciplinary intensive diabetes education programme. *Journal of Evaluation in Clinical Practice, 11,* 293-303.

King, H., Aubert, R. E., & Herman, W. H. (1998). Global burden of diabetes, 1995-2025: Prevalence, numerical estimates, and projections. *Diabetes Care, 21*, 1414-1431.

Lustman, P. J., Anderson, R. J., Freedland, K. E., de Groot, M., Carney, R. M., & Clouse, R. E. (2000). Depression and poor glycemic control: A meta-analytic review of the literature. *Diabetes Care, 23*, 934-942.

Lustman, P. J., & Clouse, R. E. (2002). Treatment of depression in diabetes: Impact on mood and medical outcome. *Journal of Psychosomatic Research, 53*, 917- 924.

Lustman, P. J., Freedland, K. E., Griffith, L. S., & Clouse, R. E. (1998). Predicting response to cognitive behavior therapy of depression in type 2 diabetes. *General Hospital Psychiatry, 20*, 302-306.

Lustman, P. J., Griffith, L. S., Freedland, K. E., Kissel, S. S., & Clouse, R. E. (1998). Cognitive behavior therapy for depression in type 2 diabetes mellitus. A randomized, controlled trial. *Annals of Internal Medicine, 129*, 613-621.

Marteau, T. M., & Bekker, H. (1992). The development of a six-item short-form of the state scale of the spielberger State–Trait anxiety inventory (STAI). *British Journal of Clinical Psychology, 31*, 301-306.

McBride, C., Atkinson, L., Quilty, L. C., & Bagby, R. M. (2006). Attachment as moderator of treatment outcome in major depression: A randomized control trial of interpersonal psychotherapy versus cognitive behavior therapy. *Journal of Consulting and Clinical Psychology, 74*, 1041-1054.

McIntyre, R. S., Fulton, K. A., Bakish, D., Jordan, J., & Kennedy, S. H. (2003). The HAM-D7: a brief depression scale to distinguish antidepressant response from symptomatic remission. *Primary Psychiatry, 10*, 39-42.

Moncher, F. J., & Prinz, R. J. (1991). Treatment fidelity in outcome studies. *Clinical Psychology Review, 11*, 247-266.

Moussavi, S., Chatterji, S., Verdes, E., Tandon, A., Patel, V., & Ustun, B. (2007). Depression, chronic diseases, and decrements in health: Results from the world health surveys. *The Lancet, 370,* 851-858.

Neff, K. D. (2003). The development and validation of a scale to measure self-compassion. *Self and Identity, 2*, 223-250.

Perepletchikova, F., Hilt, L. M., Chereji, E., & Kazdin, A. E. (2009). Barriers to implementing treatment integrity procedures: Survey of treatment outcome researchers. *Journal of Consulting and Clinical Psychology, 77*, 212-218.

Piet, J., Hougaard, E., Hecksher, M. S., & Rosenberg, N. K. (2010). A randomized pilot study of mindfulness-based cognitive therapy and group cognitive-behavioral therapy for young adults with social phobia. *Scandinavian Journal of Psychology*. Published Online: Feb 23 2010, DOI: 10.1111/j.1467-9450.2009.00801.

Polonsky, W. H., Anderson, B. J., Lohrer, P. A., Welch, G., Jacobson, A. M., Aponte, J. E., et al. (1995). Assessment of diabetes-related distress. *Diabetes Care, 18*, 754-760.

Radloff, L. S. (1977). The CES-D scale: A self-report depression scale for research in the general population. *Applied Psychological Measurement, 1*, 385-401.

Raes, F., Dewulf, D., Van Heeringen, C., & Williams, J. M. (2009). Mindfulness and reduced cognitive reactivity to sad mood: Evidence from a correlational study and a non-randomized waiting list controlled study. *Behaviour Research and Therapy, 47*, 623-627.

Resnick, B., Bellg, A. J., Borrelli, B., DeFrancesco, C., Breger, R., Hecht, J., et al. (2005). Examples of implementation and evaluation of treatment fidelity in the BCC studies: Where we are and where we need to go. *Annals of Behavioral Medicine: A Publication of the Society of Behavioral Medicine, 29,* 46-54.

Russo, S., Kema, I. P., Bosker, F., Haavik, J., & Korf, J. (2009). Tryptophan as an evolutionarily conserved signal to brain serotonin: Molecular evidence and psychiatric implications. *The World Journal of Biological Psychiatry: The Official Journal of the World Federation of Societies of Biological Psychiatry*, *10*, 258-268.

Schwarzer, R., Diehl, M., & Schmitz, G.S. (1999). The Self-Regulation Scale. Berlin: Freie Universitat, Gesundheitspsychologie.

Segal, Z. V., Teasdale, J. D., & Williams, J. M. (2002). *Mindfulness-based cognitive therapy for depression: A new approach to preventing relapse*. New York: Guilford Press.

Shigaki, C. L., Glass, B., & Schopp, L. H. (2006). Mindfulness-based stress reduction in medical settings. *Journal of Clinical Psychology in Medical Settings, 13*, 209-216.

Spitzer, R.L., Kroenke, K., Williams, J.B.W., & Lowe B. (2006). A brief measure for assessing generalized anxiety disorder - the GAD-7. *Archives of International Medicine, 166*, 1092–7.

Stoppelenburg, E. (2008). Effecten en waardering van de cursus mindfulness based cognitieve therapie [outcomes and appreciation of the course mindfulness based cognitive therapy]. *GGzet Wetenschappelijk, 12.*

Thomson, J., Rankin, H., Ashcroft, G.W., Yates, C.M., McQueen, J.K., Cummings, S.W., (1982). The treatment of depression in general practice: a comparison of L-tryptophan, amitriptyline, and a

combination of L-tryptophan and amitriptyline with placebo. *Psychological medicine*, *12*, 741–51.

Trapnell, P. D., & Campbell, J. D. (1999). Private self-consciousness and the five-factor model of personality: Distinguishing rumination from reflection. *Journal of Personality and Social Psychology, 76*, 284-304.

van der Ven, N. C., Hogenelst, M. H., Tromp-Wever, A. M., Twisk, J. W., van der Ploeg, H. M., Heine, R. J., et al. (2005). Short-term effects of cognitive behavioural group training (CBGT) in adult type 1 diabetes patients in prolonged poor glycaemic control. A randomized controlled trial. *Diabetic Medicin : A Journal of the British Diabetic Association, 22*, 1619-1623.

Vervaeke, G., & Vertommen, H. (1993). De werkalliantie: Visies op een bruikbaar concept en de meting ervan. *Tijdschrift Voor Psychotherapie, 19*, 2-16.

Wei, M., Russell, D. W., Mallinckrodt, B., & Vogel, D. L. (2007). The experiences in close relationship scale (ECR)-short form: Reliability, validity, and factor structure. *Journal of Personality Assessment, 88*, 187-204.

Wells, A., & Davies, M. I. (1994). The thought control questionnaire: A measure of individual differences in the control of unwanted thoughts. *Behaviour Research and Therapy, 32*, 871-878.

Wild, S., Roglic, G., Green, A., Sicree, R., & King, H. (2004). Global prevalence of diabetes: Estimates for the year 2000 and projections for 2030. *Diabetes Care, 27*, 1047-1053.

World Health Organization: Regional Office for Europe (1998) Well-Being measures in primary health care: The DepCare Project. Consensus meeting, Stockholm

Zautra, A. J., Davis, M. C., Reich, J. W., Nicassio, P., Tennen, H., Finan, P., Kratz, A., Parrish, B., & Irwin, M. R. (2008). Comparison of cognitive behavioral and mindfulness meditation interventions on adaptation to rheumatoid arthritis for patients with and without history of recurrent depression. *Journal of Consulting and Clinical Psychology, 76*. 408-421.
